# Developing an Integrated VR Infrastructure in Architectural Design Education

**DOI:** 10.3389/frobt.2020.495468

**Published:** 2020-10-22

**Authors:** Serdar Aydin, Begüm Aktaş

**Affiliations:** ^1^Department of Architecture, Mardin Artuklu University, Mardin, Turkey; ^2^Department of Architecture, Istanbul Technical University, Istanbul, Turkey; ^3^Department of Architecture, Altınbaş University, Istanbul, Turkey

**Keywords:** virtual reality, student-centered design education, digital design ecosystem, interaction, design-research, user experience, representational ecosystem, design skills

## Abstract

With the advent of computer technology, Virtual Reality (VR) became an integral part of design studios in architecture education. Researchers have been exploring how VR-enhanced design studios can be assessed from a student-centered perspective. This paper illustrates the role of teaching architectural design for developing a novel and contextual curriculum based on an analysis of student feedback. The background focuses on the development of VR-based architectural design education. The methodology frames two digital design ecosystems which are experimented in four undergraduate courses. With an ecosystem-based approach discussed in this paper, a medium-oriented and a content-oriented curriculum are offered for testing students' reaction to teaching design in VR. In both ecosystems, students are engaged with advanced digital design methods and techniques, which include 3D form-finding, building information modeling, visual programming, coding, and real-time rendering. The study screens the usage of software solutions for the creation of complex virtual environments, covering Blender, Rhinoceros, Unity, Grasshopper, and Revit. The implementation of a User Experience Questionnaire (UEQ) comparatively demonstrates the performative qualities of both digital design ecosystems. Results indicate that the intensity of interaction varied in two incomparable, but connate, levels of qualities. The findings suggest that the perspicuity aspects of student interaction bare the risk of “complicated” and “confusing” software. The results further demonstrate a conflict between task-related qualities and non-task related qualities. Additionally, interacting with VR tools in architecture design education is found attractive, stimulating, and original despite low scores on the pragmatic qualities of perspicuity, efficiency, and dependability. The data and results obtained from this study give insight into the planning of design studios in architecture education based on the use of VR and digital methods. Therefore, this study contributes to future research in the contextualization of the design teaching efforts.

## Introduction

Virtual Reality (VR) has made profound impacts on architectural design education. VR practices of architecture students include digital 3D modeling, realistic visualization, material and environmental simulation, and remote collaboration, which move the domain of architecture beyond conventional boundaries. Despite the widespread use of VR applications in architectural design, teaching in VR confront the issues of integration with the curriculum and of qualification standards, which vary between architecture programs taught in different countries. Extra-curricular activities, such as workshops and online tutorials, provide supplementary learning environments, whereas many architecture schools have to face the challenge to change their curricula which have been persistently applied for years. Lack of competent staff, who are qualified to teach design in VR, may count one of the causes for the endurance of conventional teaching environments. Yet, especially after the COVID-19 pandemic, which affected architecture studies greatly, it is realized how important creating an infrastructure for teaching architectural design in VR.

For many years, researchers, who are dedicated to finding out how digital instruments can improve architecture education, have made significant contributions to the current willingness for architecture education being viable online. Studies include the impact of VR and digital instruments in design creativity (Alvarado and Maver, 1999; Oxman, 2008; Celani, 2012; Shih et al., 2017; Coppens et al., 2018), design representation (Indraprastha and Shinozaki, 2009; Pelosi, 2010; Felbrich et al., 2018) and design communication (Dorta et al., 2016a,b; Schnabel et al., 2016). The recent research aggregate interest in examining student-centered and user-oriented approaches through qualitative (Kreutzberg, 2014; Gül and Kilimci, 2017) and quantitative methods (Wang et al., 2019). Also, medium-centered approaches are conducted to unravel the impact of VR tools as Digital Design Ecosystems (DDEs) (Al Bondakji et al., 2018).

On the students' side, digital design ecosystems progressively evolve from the creation of simple virtual environments. One of the key elements in student design workflows is the ability to connect and shift between different VR environments and tools. Although the connectivity of VR environments and tools is highly important, cognitive abilities, skillsets, mindsets, and thinking are other crucial factors for measuring the impact of VR. In other words, design environments should provide all the useful tools, that design learners, as well as practitioners, need at the right time, rather than being limited to specific design media (Shih et al., 2017). There is an increasing interest in conceptually comprehending such environments that this paper attempts to identify as Digital Design Ecosystems (DDEs). Researchers argue that the DDEs include a range of aspects that could help us understand the role of VR in design studios (Davis, 2013; Rogers and Schnabel, 2018). In this work, the benchmarks of teaching architectural design in VR to measure the quality of efforts include attractiveness, perspicuity, efficiency, dependability, stimulation, and novelty. In the present study, the role of teaching architectural design via VR tools in an interactive multimedia environment that is demonstrated through a combination of student-centered and technology-oriented study that aims for developing a contextual VR curriculum at the Department of Architecture^1^, Mardin Artuklu University, which is located in the southwest city of Mardin in Turkey.

## Related Works

The evolution of computer graphics has been instrumental for early technologists to circulating VR tools in the domain of architecture. Sutherland's (1963) Sketchpad, which was described as “a man-machine graphical communication system,” is acknowledged to be one of the most influential inventions for architectural design, practice, and education (Salim and Burry, 2010; Davis, 2013). Sketchpad was not only a software but also a hardware solution for architecture. VR infrastructures that integrate hardware and software solutions address creative learning in architectural design education, which entails a sense of inclusion, navigation and manipulation (Helsel, 1992). Likewise, we can classify the proceeding applications and studies in architecture, according to their focus on hardware or software. On the one hand, hardware-based VR projects allowed the transformation of exclusive VR technologies into affordable tools for interactive, responsive, and immersive design solutions, such as Sensorama (Heiling, 1962), The Ultimate Display (Sutherland, 1965), GROPE (Batter and Brooks, 1972), Glowflow (Krueger, 1977), Videoplace (Krueger and Wilson, 1985), VIVED (NASA, 1988), VPL DataGlove (Lowood, 2019), Fake Space Labs' BOOM (Mazuryk and Gervautz, 1999, p. 24), and Virtual Wind Tunnel (Bryson, 1993). On the other hand, software-based VR evolvement in applications contributed to architectural learning in novel digital settings (Donath et al., 1999; Oxman, 2006). The two categories of research contribute to the current needs from digital design environments which are sufficiently interactive, responsive and immersive.

Since the early developments on hardware and software, the use of VR tools have evolved from basic operations to advance solutions for the creation of virtual environments, which include components of 3D models, dynamic real-time renderings, closed-loop interactions, and even enhanced sensory feedback (Wickens, 1992; Kalay, 2004; Schnabel, 2009; Sorguç et al., 2017). Further, design educators accommodate novelties of VR for reinforcing teaching methods that aim for developing cognitive abilities (Tokman and Yamacli, 2007; Schnabel and Howe, 2010; Gül and Simisic, 2014; Shih et al., 2017).

As it appears that design is a highly cognitive process, VR-based teaching models require developing student-centered approaches which stem from the need for understanding the impact of novel design environments. In the present study, the use of VR in architectural design education is reviewed through two distinguished stages that cover the last three decades of studies, Virtual Design Studios (VDSs) and Digital Design Ecosystems (DDEs).

### Design in Virtual vs. Real

**Figure 1:**
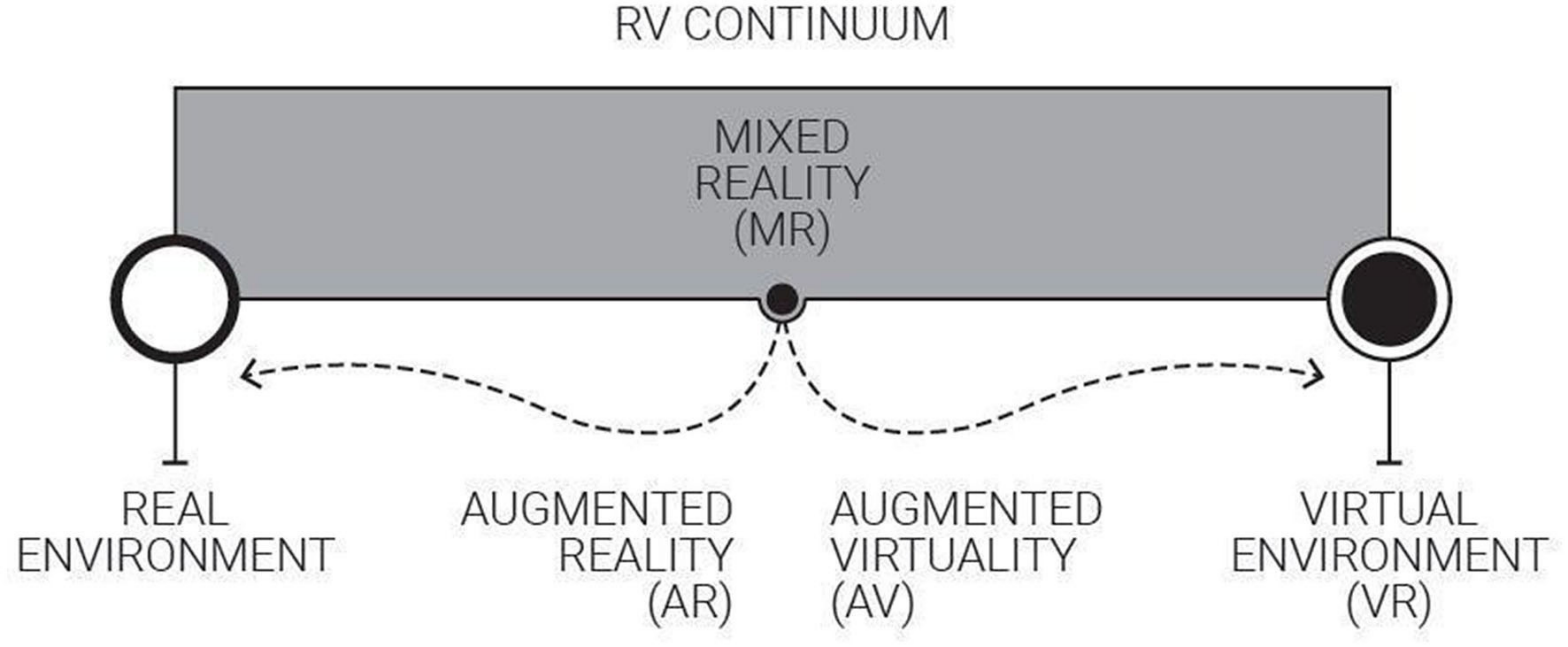
Diagram of the Reality-Virtuality (RV) continuum. Adapted from Milgram and Colquhoun (1999).

Architectural design education is mostly based on visual expressions. Communication with visual expressions and representations is subject to the constant change in order to meet the understanding and appreciation of actors involved in design processes (Coyne, 1994). In virtual environments, physical expressions transcend from gravity-constrained reality into virtuality imitating gravity (Kotnik, 2010; Naz et al., 2017). Since the retrieval period of VR as new technology from computer science, the Reality-Virtuality (RV) continuum (Milgram and Colquhoun, 1999) has been a remarkable diagram for the VR scholarship in computer-aided architectural design (CAAD) (see Figure 1). The diagram is “limited strictly to visual displays,” setting the real and the virtual in contrast (Milgram et al., 1995). Conformably, for about two decades, the incentive of VR in design studios has remained limited to the appreciation of display properties. From a “designerly” point of view (Cross, 2006), teaching architectural design in VR should be more than a display technology as design activities often require to transcend the limits of the design tool.

In response to the understanding of VR as a linear diagram extending from reality to virtuality, Schnabel et al. (2004) articulates a responsive boundary between these two realms. Based on Schön's (1983) theory of design as a reflexive practice, Schnabel et al. (2004) do not take for granted the duality between reality and virtuality but utilize digital and virtual environments as part of a design ecosystem in which real and virtual activities engage in symbiosis. Yet, the creation of a sense of place in VR is viewed through computer-mediated dynamic worlds (Gu and Maher, 2005). In studios, students engage with a physical and digital medium for imaginary scenarios requested in design briefs. Within this context, Gero and Kannengiesser (2012) define that one of the goals of design research is to better understand the ways in which end-users interact with the products of designs. In this regard, the present study is not exclusively dedicated to displaying qualities of immersive VR applications. This paper delineates two ecosystems that reflect the experience of students, instead.

### Virtual Design Studios (VDSs)

Early efforts to bring VR into architecture education include experiments in Virtual Design Studios (VDSs). In search of innovation in the 1990s, researchers focused on measuring the impact of VDSs in creativity (Bradford et al., 1994; Achten et al., 1999). VDSs are admittedly reported to move design education beyond conventional boundaries and curricula. The positive impact of VDSs include new modes of participation from diverse professional fields. Alternative concepts include Social Network VDS (Schnabel and Ham, 2011) and Interprofessional VDS (Schnabel and Howe, 2010) that prove new types of collective authorship (Kolarevic et al., 1998).

In parallel, the concept of VDS advanced from a range of tools, systems, and services, which help replicate, simulate or supplement the conventional design studio (Jones et al., 2017), into environments for advanced 3D modeling, analysis, and fabrication (Lynn, 1999; Peters and De Kestelier, 2008; Adel et al., 2018). Additionally, the recent works that take advantage of VDS deal with real-time and dynamic rendering (Gün, 2013), generative and parametric processes (Felbrich et al., 2018; Newnham et al., 2018), responsive and interactive solutions (Fox, 2009; Beesley et al., 2010), and sensory feedback loops (Ahlquist, 2016; Moleta et al., 2018).

## Concept and Method

### Mapping Interaction: Digital Design Ecosystems (DDEs)

Architectural design education takes place through interactive studio environments. In studios, students make reciprocal interchange of ideas between physical and digital media for the creation of imaginary scenarios that respond to design briefs. Monitoring students' learning process naturally become a significant element of complex design ecosystem which evolves from the use of virtual environments and digital tools. To increase the level of engagement with the use of VR tools and environments, complementary courses and online tutorials help students learn digital design methods. However, at the end of design processes, a student's progress is assessed without necessarily evaluating design learning processes in relation to VR and digital tools which are mostly learnt outside the studio. This study provides a holistic approach to evaluate digital design ecosystems that differ in the use of digital and virtual media. Digital design ecosystems can be reviewed by looking at the medium, the user, and the content as proposed in related disciplines, such as interactive and participatory design (Sterk, 2006; Maher et al., 2007), new media (Murray, 1998), and cybernetics (Bermudez, 1997; Fischer, 2017).

In this research, interlinked connections of VR tools, students and contents help map out the basic elements of the digital design ecosystem under discussion. In the present study, there are two models of ecosystems, which are defined by the type of teaching and learning activities in relative courses. Ecosystem-I is based on medium-oriented, and Ecosystem-II is on content-oriented design activities. The former privileges the teaching of software tools for problem-solving, whereas the latter stresses the production of design research outcome while finding the problem. Based on the systematization of DDEs among medium, student and content, this paper deploys a User Experience Questionnaire (UEQ) that ascertains the determinant role of the student, for understanding how an integrated VR infrastructure can be developed contextually at Artuklu Architecture.

#### Context

The architectural design education at the Artuklu Architecture currently faces the issues of international recognition, limited budget and the lack of space and workshop facilities including VR lab instruments. The school has 6 generations of undergraduate alumni as it is a decade old. In 2010, the Artuklu Architecture was founded on a heritage context in an ancient town where the premises of the school is a historical building. Accordingly, the research focus has intensely been on architectural history as part of the strategic targets of the university leaning toward social sciences. Despite negative circumstances, design teaching has been open to new ideas and improvement to follow international standards. The national recognition of Artuklu Architecture can be measured by the number of student awards rewarded to Artuklu Architecture through national student design competitions (ArcED, 2019).

In this institutional context, it requires a well-structured contextual program to invite and encourage students to learn how to use cutting-edge digital and VR technologies by which international standards of architecture education are addressed (Akin, 1990; Alvarado and Maver, 1999; Kvan, 2004). To invest in advanced digital tools, the formulation of an integrated infrastructure plays an important role in design studios. Pursuing an integrated program is likely to produce sustainable iterations and successful results in parallel with the technological evolution of design studios. As such, risks and legal processes are involved in financing an integrated VR infrastructure at a young school like Artuklu Architecture. By that, this paper directly contributes to the progress of design education at the Artuklu Architecture while offering a real case for other architecture schools having similar circumstances. Besides, the paper exhibits results that can be compared with the adaptation of VR tools in schools worldwide.

#### Courses

In this study, four architecture courses taught at the Artuklu Architecture are subject to measure the impact of teaching architectural design in VR. The study presents the usage of digital design tools by students with limited to no-experience in prior. The four courses are; Architectural Studio II, Computer Based Design and Representation II, Architectural Studio VI and Digital Heritage and Design. The courses studied in this paper belong to the second term (spring) in the 2018/19 academic year. Although some of the andragogy aspects are in line with each other, the courses vary in their teaching format. In this study, they are classified as medium-oriented (Architectural Design Studio II and Computer-based Design and Representation) and content-oriented courses (Architectural Studio VI and Digital Heritage and Design) (see Table 1). Later, the paper will discuss two ecosystems created by the classification of courses and the relative andragogy aspects.

**Table 1:** VR courses and corresponding design ecosystems.

**Course code**	**Course name**	**Teaching format (weekly)**	**Software**	**Digital design tools**	**VR design ecosystem**
MIM 102	Architectural Studio II	2 h lecture 2 h tutorial 4 h studio	Blender and Rhinoceros	3D modeling	Ecosystem I: medium-oriented
MIM 206	Computer Based Design and Representation	1 h lecture 3 h tutorial	Revit and Rhinoceros	3D modeling and documenting	
MIM 302	Architectural Studio VI	2 h tutorial 6 h studio	Rhinoceros and Grasshopper	Advanced 3D modeling via visual programming language	Ecosystem II: content-oriented
MIM 444	Digital Heritage and Design	1 h lecture 1 h tutorial	Unity	Real-time virtual engine	

Both ecosystems include studio-based and non-studio-based courses. In all courses, the director drives the process of a number of students by giving feedback on individual design tasks or assignments. Several andragogy aspects of these courses can be underlined. On the one hand, the roles of the course director are instructive for the selection of design medium (instructor), indicative for the design decisions (indicator) and informative for the development of students' proficiency (supporter). On the other hand, a student has to take a few roles to deal with the requirements of the course. For the selection of design media, the student is mostly an explorer who follows maps of information given by the instructor but they are free to choose any design tool and medium. For decision making, the student is required to be an adventurer who would be intuitive for creativity and innovation. As for individual proficiency, every student has to be determined to develop new design skills. The summary of the roles (Table 2) presents the connection between both sides of roles, i.e., of the course director and student. In the presented course, the roles need to be responsive in order to arrive at acceptable results which are expected and outlined at the beginning of the semester.

**Table 2:** Summary of the roles of the course director and the student models in line with andragogy categories.

	**Andragogy categories**	**Course director**	**Student**
1	Selection of design tools/media	Instructor who encourages the use of new tools	Explorer who is ready to learn new tools in order to follow novel design processes
2	Decision making to develop design content	Indicator who expects the student to find creative ways	Adventurer who is ready to find creative solutions for given tasks
3	Individual development	Supporter who informs ways for individual development	Pioneer who is ready to develop new design skills with advance design tools

As the table indicates above, the courses are built on andragogic ways of teaching which offer a map for a student-oriented approach. The impact of the andragogy aspects is context-dependent and may vary between different circumstances faced by students. To sustain and improve the student-centeredness in the andragogy aspects, two general surveys are conducted at the beginning and the end of the courses. General surveys were done to receive student feedback and suggestions about the expectations and the performances of both themselves and the course directors. But these general surveys are excluded from the present study, only targeting a user experience questionnaire which generates data for this study as explained later.

### Current Framework

Each course had its own online learning platform to discuss and share course-related ideas, concerns, and solutions. Two of these courses (Architectural Design Studio II and Computer-based Design and Representation) focused on learning 3D modeling methods to create virtual environments while improving skills for using the software. The other two courses (Architectural Studio VI and Digital Heritage and Design) were led by design-research teaching objectives to drive students toward creative design content. The outcome of the courses differs in the duration that takes for design ideation, the level of complexity, the scale of a design problem, the variety of scales for blueprints and other parameters that are taken into account. Each course includes the teaching of specific digital design methods, whereas only an acceptable level of knowledge was enough but not required. The digital design methods cover specific software, i.e., Blender, Rhinoceros, Grasshopper, and Unity. In Architectural Design Studio II, 3D modeling tools, Blender, and Rhinoceros, are taught to model complex geometries. In Computer-based Design and Representation, second-year students learn 3D design by using the concept of Building Information Modeling (BIM). Architectural Studio VI is an architectural design studio that relies on a variety of 3D design tools enhanced with visual programming languages, preferably Rhinoceros with Grasshopper. As for Digital Heritage and Design, a real-time virtual engine, Unity, is used based on the objective of digitalizing heritage environments.

### Measuring Interaction: Student-Centered Design Education

In architecture education, students develop individual and contextual study environments. The proposed method examines the development of a contextual Digital Design Ecosystem (DDE) by mixing quantitative and qualitative approaches.

In this study, we deploy the UEQ to first measure the quality of teaching architectural design in VR from the viewpoint of students. The UEQ is designed to help us understand how successful interactive experiences are (Rauschenberger et al., 2013; Schrepp et al., 2014). It measures the scales of attractiveness, perspicuity, efficiency, dependability, stimulation, and novelty of designed experiences in 26 questions. Each scale is assessed by the user with a value between 1 and 7, representing opposing pairs of scales.

Likewise, the benchmarks of teaching architectural design in VR may include attractiveness, perspicuity, efficiency, dependability, stimulation, and novelty. At the end of the courses, a survey with 26 questions was conducted, based on voluntary participation. The survey asked students to assess their experiences with VR design tools. The number of respondents is 29, 26, 8, and 14 for Architectural Design Studio II, Computer-based Design and Representation, Architectural Studio VI and Digital Heritage and Design, respectively. In total, 75 responses were collected, which are combined with semi-structured interviews with students. This study then synthesizes the quantitative and qualitative results for evaluating the contribution of VR tools to the production of architectural design at different levels.

## Ecosystem I: Medium-Oriented Design in VR

### Procedure

In Ecosystem I, students are exposed to a package of 3D tools for design, modeling, documentation, rendering, and animation, which are Blender, Rhinoceros, and Revit. It is required to resolve given design problems based on the creation of 3D virtual environments and 2D drawings. During the semester, the student work and performance are discussed in design reviews and one-to-one discussions. Having been tracked as part of the medium-oriented design process, the outcome is graded individually.

In general, we should underline several aspects of the teaching procedure of the courses from both students' and instructor's point of view. From the instructor's points of view, what specific modeling tools that students should practice following the course syllabus is determined at the beginning of the term, which forms the basis of the medium-oriented ecosystem. Within the scope of this course, Blender, Rhinoceros, and Revit are utilized and practiced. From the students' perspective, these tools (software) are relatively harder software for them. Students were encouraged about the use of these specific modeling tools that meet the criteria of the course. Two courses are studied in Ecosystem I, and below are given more details on how each course is conducted.

#### First-Year Students' Course: Architectural Design Studio II

The first-year design studio (Architectural Design Studio II) is a course that offers basic architectural design principles. The learning objectives of the course include understanding and communicating architectural design ideas with 2D and 3D representation methods, gaining individual, and collaborative skills to resolve spatial problems and complexities. According to the course format, students are grouped within the range of instructors while following an additive learning environment of learning new techniques and knowledge of architecture.

Eventually, 80 students went through a set of given design problems. Three design briefs were asked from students to resolve. The first brief was about redesigning a dream seen in the past, using 2D Manga storytelling and visualization techniques. The second was the 2D mapping of urban glitches found in the ancient city of Mardin and then turning it into 3D animation. The last but the longest one was the design of a living habitat in the context of a small village near Mardin. Students were introduced how to use Blender and Rhinoceros for modeling 3D virtual environments. The assessment in Architectural Design Studio II is conducted through an independent jury at the end of the semester.

#### Second-Year Students' Course: Computer-based Design and Representation

In the second-year course (Computer-based Design and Representation), digital design methods are taught. The learning objectives of the course include creating complex geometries and architectural forms by means of 3D digital modeling techniques, obtaining knowledge about computational design and designing virtual environments to discuss design issues in complex geometries. The course format is made of weekly courses, weekly assignments, a design project, computer lab studies and the use of online learning platforms on Google Groups and Facebook.

In this compulsory course, students were given a three-phase study form that varied from low-poly voxel-based modeling to BIM, and advanced modeling. Each phase is identified with the tools (Revit and Rhinoceros) which are used to create 3D models in their environments. The first one was based on voxel representation that limited, yet sped up, the process of design ideation while realizing 3D environments. The second phase focused on conceptual 3D model making through constrained and parameterized geometries. The last phase was about transferring the geometries from the previous software into a modeling environment through free-form geometries. Theoretical classes supported and encouraged the process of convincing students who had a lack of knowledge about why specific software is needed to be learned to design better in virtual reality. A brief history of CAAD was provided during lectures after which students followed hands-on tutorials every week. Along with 7 weekly assignments, a final design project was submitted in a design portfolio which marked the end of the course requirements to pass. The assessment items measure learning modules collected from weekly assignments, a final project and a portfolio design.

### Participants

The compulsory courses of Architectural Design Studio II and Computer-based Design and Representation were conducted during the Spring semester in 2019, with 80 and 49 students enrolled, respectively. Survey participants were recruited using e-mail and social media groups dedicated to each course. No demographic, gender and age data were collected as the course level defines the limits of the present study.

### Results and Analysis

**Figure 2:**
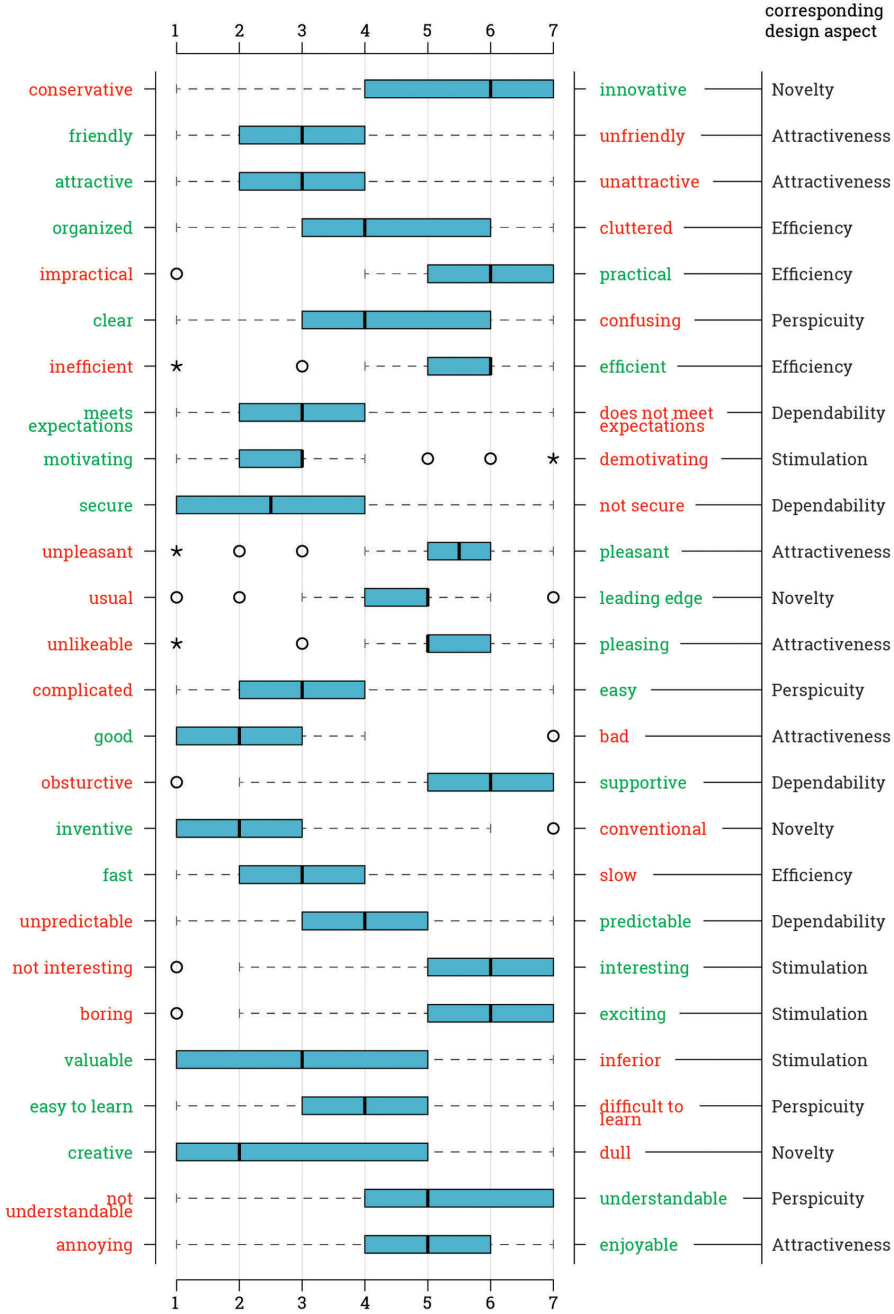
Answer distribution per questionnaire item for the Ecosystem I, i.e., medium-oriented.

Students were asked the 26 questions of UEQ relating interaction in 6 different aspects. The order of the questions was random and the evaluation was based on a 1-to-7 Likert scale. For some questions, the left end of the scale represented a positive design characteristic, such as clear and organized, whereas some questions had a positive value on the other side. Regarding Ecosystem I, the most notable negative values are from “complicated/easy” (*M* = 3.14, *SD* = 1.38) and “clear/confusing” (*M* = 4.56, *SD* = 1.88), which are related to perspicuity. Results suggest that out of 26, 25 questions are marked either neutral or positive on average (Figure 2).

**Figure 3:**
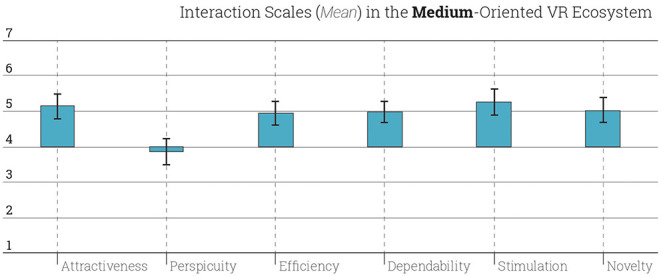
Interaction scales scored in Ecosystem I. Error bars represent the 95% confidence intervals.

The UEQ has correspondent design aspects for each questions. It is found that the design aspects of attractiveness (*M* = 5.15, *SD* = 1.32), efficiency (*M* = 4.95, *SD* = 1,24), dependability (*M* = 4.99, *SD*= 1.15), stimulation (*M* = 5.26, *SD* = 1.33), and novelty (*M* = 5.03, *SD* = 1.31) show tendency for a successful ecosystem. However, students found the perspicuity (*M* = 3.87, *SD* = 1.37) of Ecosystem I is much lower than the rest of the aspects (Figure 3).

**Figure 4:**
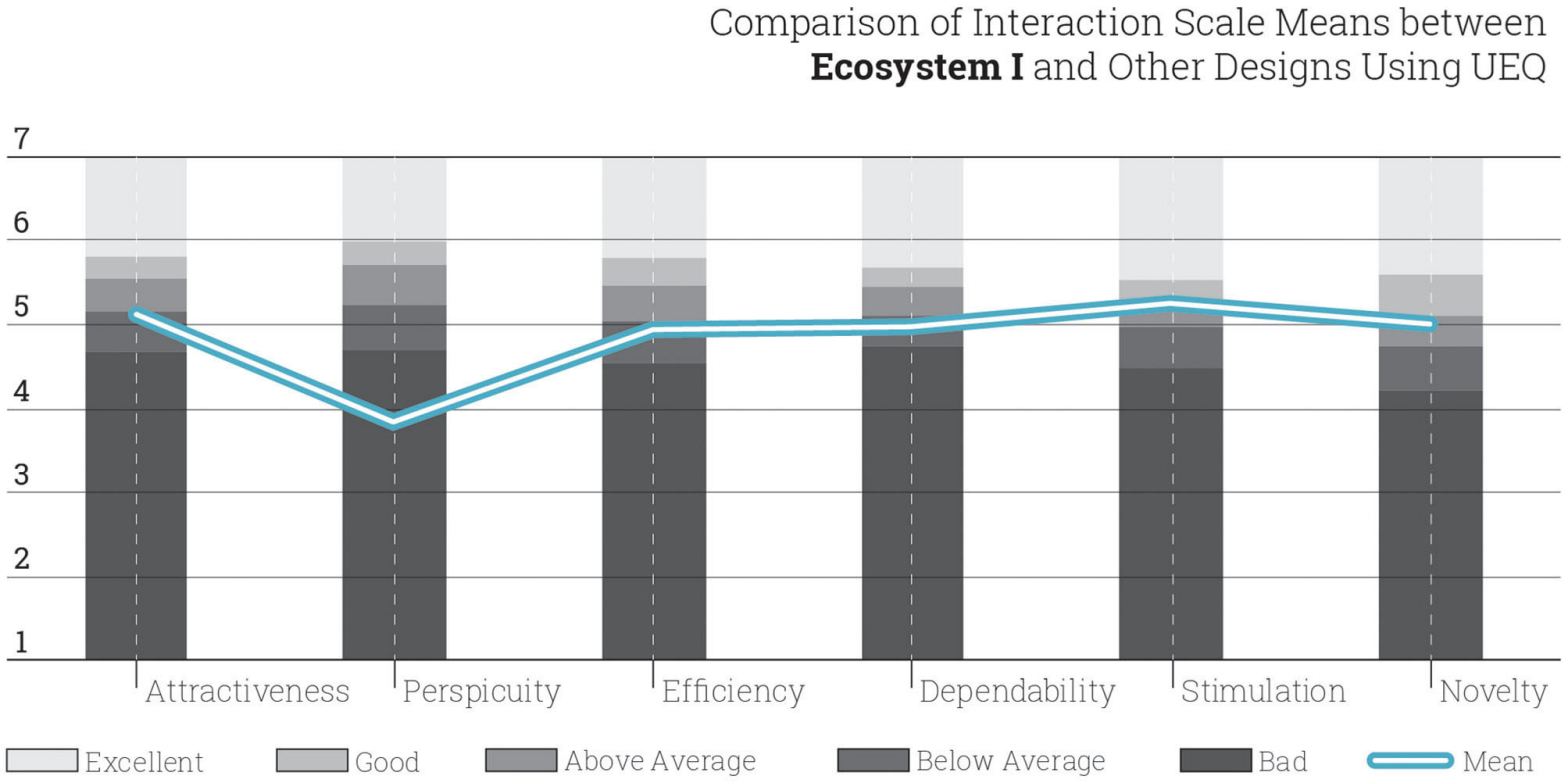
Comparison of student interaction in Ecosystem I with other design products.

In comparison to data created by other design products that are evaluated with UEQ, the performance of Ecosystem I is bad in perspicuity; below average in attractiveness, efficiency and dependability; and above average in stimulation and novelty. This means that the Ecosystem I and the VR tools used here are in the range of the 25% worst results among all UEQ-evaluated design products (Figure 4). It is worthwhile to note that the perspicuity aspect in Ecosystem I tends to be not good not only when compared to the other aspects but also other products and systems. This shows that the VR tools applied the courses of Ecosystem I were too complex and the learning curve was steep. According to the comparison of the data through *t*-test, there was a significant difference for the perspicuity (*M* = 3.87, *SD* = 1.37) conditions; *t*_(53)_ = 0,027, *p* = 0.05. In that sense, tools and software which were learned and deployed in Ecosystem I were harder for students than expected although these the medium-oriented courses are attractive, stimulating, and novel for the students.

Perspicuity is one of the pragmatic qualities which includes efficiency and also dependability. Despite the low score in perspicuity, in sum, Ecosystem I can be categorized as just above average for its task-related qualities. Therefore, perspicuity of Ecosystem I should be specifically evaluated for further studies. Also the non-task related hedonic qualities, i.e., stimulation and originality, of the Ecosystem I appears to be good.

## Ecosystem II: Content-Oriented Design in VR

### Procedure

In Ecosystem II, students are, first of all, expected to generate creative content that can provoke substantial discussions about the given design problem. In parallel, digital tools (Unity, Rhinoceros, and Grasshopper) are taught via tutorials, hands-on experiments and the online learning and sharing platforms dedicated to each course. Two main (mid-term and final) and three interim design reviews are organized for each course. The main motive in Ecosystem II is to engage students with the computational aspects of the design requirements. In effect, the concentration on computational aspects greatly determines the shape of the design process and outcome. In this context, the courses aim at characterizing how students demonstrate their design-research ideas based on architectural knowledge and creativity.

Similar to Ecosystem I, here are two courses studied to examine the effects of Ecosystem I on teaching design with digital instruments. The instructive role of the course director is defined to be more encouraging for exploring new tools, instead of specific ones. Students had more freedom for using the opportunity to explore many digital tools, whereas the learning process is supported via tutorials and hands-on experiments. However, in this case, the selection of tools (Rhinoceros with Grasshopper and Unity) was concentrated on the level of popularity in architecture rather than efficiency to be tested by students. Therefore, in this study, we cannot measure the impact of other tools that may otherwise be part of Ecosystem II because it is content-oriented. The details about the courses are provided below to shed light on the background of Ecosystem II.

#### Third Year Students' Course: Architectural Studio VI

The third-year design studio (Architectural Studio VI) is offered to a group of 15 students. The learning objectives are based on the concept of “digital spontaneity” that seeks computational design solutions for urban problems. The studio develops through a design-research process embedded in weekly workshops, tutorials, design reviews, individual studies, presentations, digital lab applications, and virtual implementations as well as the use of online learning platforms as Google Groups, Pinterest, and Instagram. The course calendar is made of 4 milestones; research and analysis, conceptual design, developed design and final outcome. Meanwhile, students have to learn digital design methods in Rhinoceros with Grasshopper environment, which they did not use before. The Architectural Studio VI studio is supported with advanced parametric design tutorials to engage the students with digital design principles in theoretical discussions as well as a certain level of practical issues. The studio requires to move between micro and macro scales while producing heterogeneous relations between topologies and geometries. Design ideation produces contextual outcomes for each individual designer. The studio is conducted in a student-centered manner, whereas the studio requirements allow flexibility in choosing methods linked with the design concept.

In this year's Architectural Studio VI design studio group, students were strongly encouraged and supported to swiftly integrate 3D modeling environments into their conventional workflow due to lack of technological skills. For this, students were given the freedom to delineate their own functionality around the theme of entertainment. The design brief did not include information about the scale and required space so that students could customize and adapt advanced digital workflows into their design ecosystem.

The assessment in Architectural Studio VI is based on the individual design process and how well the submission requirements are met. At all 4 milestones of the studio, different types of submissions are asked from students. In the research and analysis part, diagrams, drawings, models, animations, parametric, and algorithmic descriptions as well as all sorts of visual materials are expected from submission. In the conceptual design part, students present an innovative and convincing computational approach and programme with appropriate media, models, and drawings that may include a site plan, plans, sections, models, massing, pseudo-codes, scripts, animation, and parametric relationships. In the developed design stage, students prepare again all the above-mentioned media for presenting in front of an independent jury, whereas the final outcome includes the last submission that packages the all previous stages in the form of a unique story.

#### Fourth Year Students' Course: Digital Heritage and Design

The fourth course studied in this paper is an elective course and about designing interactive 3D virtual environments for the representation and digitalization of heritage in Mardin. The learning objectives include but not limited to the dissemination of digital cultural heritage via 3D models and online platforms. More specifically, students are required to develop an intermediate level of understanding about how digital glitches (or errors of virtual 3D models) can improve the interaction between the audience and the virtual heritage environments. According to the format of the course, 20 students started with individual works and later paired for the final group work.

The course started with the introduction of digital heritage and 3D photogrammetry techniques to be used for individual assignments. As for the group work, a real-time virtual engine, Unity, was introduced to create an interactive virtual heritage environment that one could enter to play like a computer game.

Student works are assessed through a final project, its storyboard and a 500-word text. As the course included lectures, students are asked to discuss the role of design in digital heritage as well as the role of the tool for the creation of interactive 3D story-telling. Despite interesting ideas and discussions made by students, these texts are not included in the present study for the evaluation of Ecosystem II.

### Participants

Digital Heritage and Design was an elective course and 20 students selected it, whereas Architectural Studio VI was compulsory and 15 students chose this group as the course was formed in 5 different design studios. Each course was conducted during the Spring semester in 2019. Students participated in the survey via e-mail and social media groups of each course. Demographic data was not required.

### Results and Analysis

**Figure 5:**
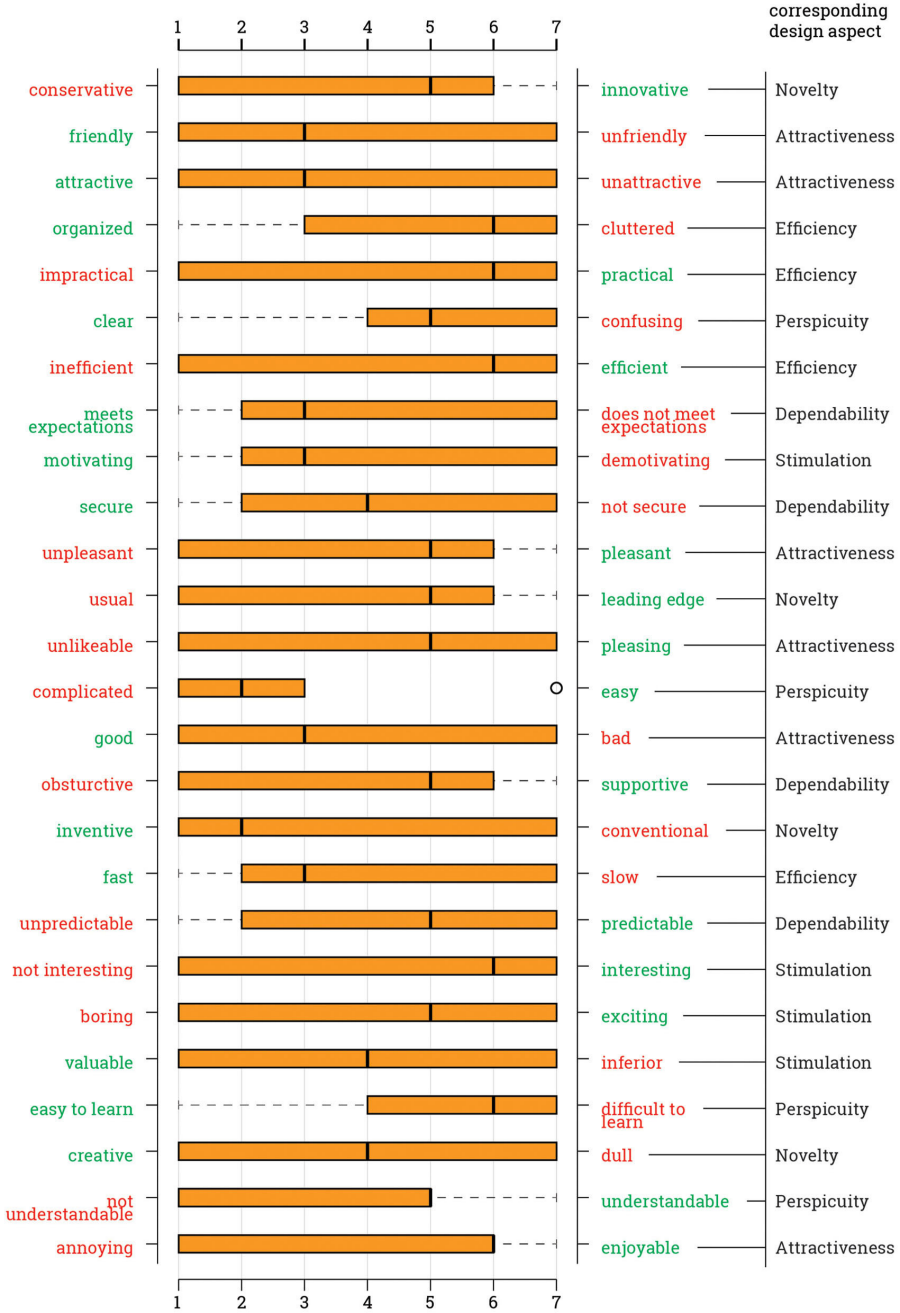
Answer distribution per questionnaire item for the Ecosystem II, i.e., content-oriented.

According to the results, Ecosystem II presents a high-level deviation (Figure 5). The reason is because of different experiences that students had in the content-oriented courses of Architectural Studio VI and Digital Heritage and Design. The analysis of this is given and discussed in the next section. Ecosystem II needs improvement in terms of “organized/cluttered” (*M* = 4.86, *SD* = 2.33), “complicated/easy” (*M* = 2.19, *SD* = 1.78), and “easy-to-learn/difficult-to-learn” (*M* = 5.00, *SD* = 2.26). Among these three, complicatedness appear to be rather evenly distributed between the two courses. The corresponding scale suggests that the rest of the 26 aspects are neutral, and there is no aspect that would explicitly indicate a high level of success. Yet, there is a tendency toward having unequivocal positive results in terms of “impractical/practical” (*M* = 4.76, *SD* = 2.68) and “inefficient/efficient” (*M* = 4.57, *SD* = 2.48) which are both about the efficiency (*M* = 4.15, *SD* = 2.23) of the ecosystem.

**Figure 6:**
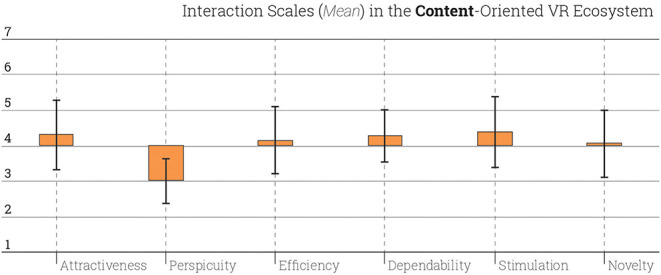
Interaction scales scored in Ecosystem II. Error bars represent the 95% confidence intervals.

Based on the UEQ result, it is found that for 21 student respondents, the mean scale for each aspect is actually below the average score compared to a collection of other UEQ studies. The error bars represent the 95% confidence intervals of the scale mean. Similar to Ecosystem I, the results indicate that the design aspects of attractiveness (*M* = 4.30, *SD* = 2.33), efficiency (*M* =4.15, *SD* = 2,23), dependability (*M* = 4.27, *SD* = 1.70), stimulation (*M* = 4.38, *SD* = 2.34), and novelty (*M* = 4.06, *SD* = 2.19) present tendency for a more successful design ecosystem. However, the perspicuity (*M* = 3.01, *SD* = 1.47) of Ecosystem II is much lower than the rest of the aspects (Figure 6).

**Figure 7:**
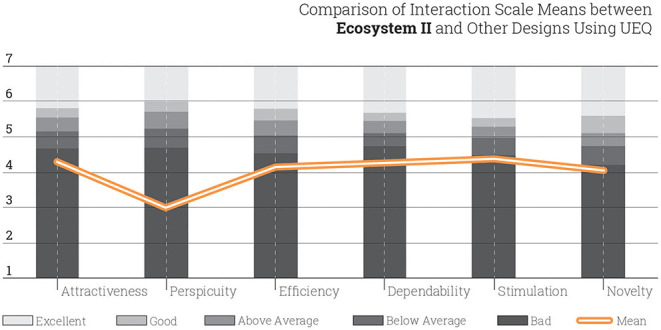
Comparison of student interaction in Ecosystem II with other design products.

When compared to other designs and systems, Ecosystem II is qualified relatively lower. However, a further inspection based on semi-structured interviews with students reveals that the learning curve of the digital tools plays a significant role in the low results generated from the survey. This shows that further study is necessary to disclose the optimal quality of teaching in Ecosystem II. It can also be inferred that, despite the low scores of Ecosystem II, the pattern of the graph is similar to the one generated for Ecosystem I. From this view, both ecosystems achieve better qualities in terms of attractiveness and stimulation when compared to other aspects (Figure 7).

The task-related pragmatic qualities, i.e., perspicuity, efficiency, and dependability, and are categorized as above average, whereas the non-task related hedonic qualities, i.e., stimulation and originality, of the Ecosystem II appear as good.

From this study, we understand that the quality of VR courses, especially Digital Heritage and Design, needs improvement, particularly in the design-research settings of DDEs. It is evident that the medium-oriented design ecosystem receives more interest in undergraduate courses. The lowest score is recorded for the perspicuity aspect of the content-oriented ecosystem, whereas the most successful aspect of both ecosystems is stimulation. In future studies, UEQ can be complemented by a pre-course survey to understand how the student reaction changes over time during the semester. Based on the UEQ results, it appears urgent to develop an alternative and integrated DDE to teach advanced design techniques via VR tools (see Table 3).

**Table 3:** Comparison of the scale means in Ecosystem I and Ecosystem II.

**Scale**	**Medium-oriented ecosystem (Architectural Design Studio II & Computer Based Design and Representation)**						**Content-oriented ecosystem (Architectural Studio VI & Digital Heritage and Design)**					
	**Mean**	**STD**	***N***	**Confidence**	**Confidence interval**		**Mean**	**STD**	***N***	**Confidence**	**Confidence interval**	
Attractiveness	5.15	1.32	54	0.35	0.79	1.50	4.30	2.33	21	1.00	−0.69	1.30
Perspicuity	3.87	1.37	54	0.37	−0.50	0.24	3.01	1.47	21	0.63	−1.62	−0.36
Efficiency	4.95	1.24	54	0.33	0.62	1.28	4.15	2.23	21	0.95	−0.80	1.11
Dependability	4.99	1.15	54	0.31	0.68	1.29	4.27	1.70	21	0.73	−0.45	1.00
Stimulation	5.26	1.33	54	0.36	0.91	1.62	4.38	2.34	21	1.00	−0.62	1.38
Novelty	5.03	1.31	54	0.35	0.68	1.38	4.06	2.19	21	0.93	−0.88	0.99

## Discussion

### Role of Instructors and Students

Responsive and interactive student experience is central to the success of teaching design and studio settings. The study gives insight into the role of the instructor and students based on the level of interaction with VR design tools. Regarding the Ecosystem II, the distribution of responses (Figure 5) shows that there may be a greater variety of student profiles that need to be considered separately. The assessment of VR design experiences could be varied due to general interest and skills acquired in prior. Depending on a matrix of the level of knowledge and learning objectives, students could be grouped at the beginning. In this regard, homogenized student teams might still lead to few borderline results. Bu strategically mixing or randomizing the groups could be more reliable. With the current results, running a reliability analysis based on Guttman's λ^2^, we see that the students mostly agree on perspicuity (λ-2 = 0.72) and dependability (λ-2 = 0.79). Although these two qualities of perspicuity and dependability show how pragmatic the ecosystem is, it is hard to evaluate because the individual mean scores of the two qualities greatly differ from each other (*M*_perspicuity_ = 3.01; *M*_dependability_ = 4.27) (Figure 7). Yet, we understand that there was an incompatibility between the given task and the learning curve of the VR tools. As for the learning curve, the language barrier was certainly a reason for Turkish-speaking architecture students as the learning resources and the utilized software are written in English. Besides, the course content could include more hands-on tutorials to cope with the pragmatic issues of the ecosystem.

**Figure 8:**
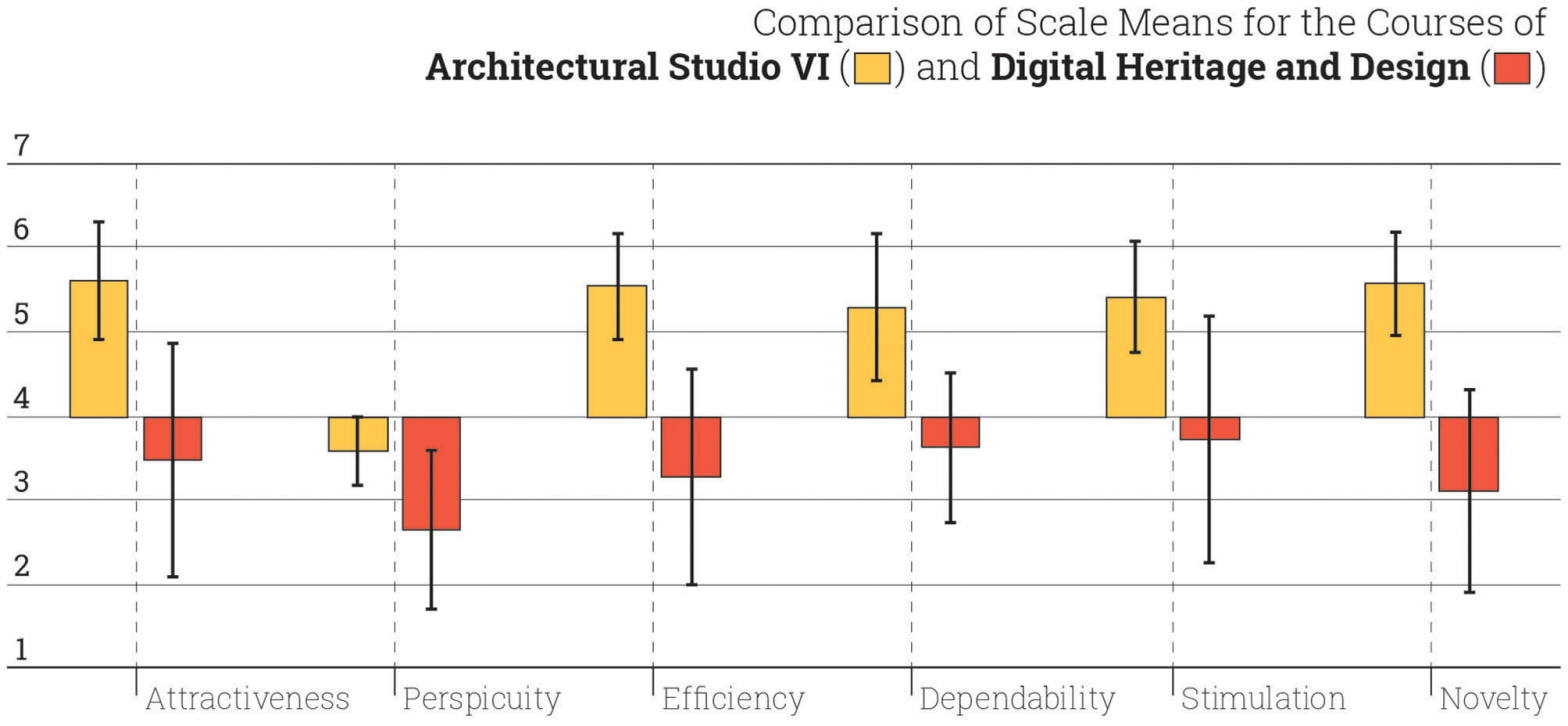
Comparison of interaction scales in Architectural Studio VI and Digital Heritage and Design.

In Ecosystem I, the instructor chooses specific instruments for students to develop 3D modeling skills. In Ecosystem II, the instruments are introduced according to the criteria that the software environment is based on a virtual programming language (VPL) for Architectural Studio VI and a real-time virtual engine (RTVE) for Digital Heritage and Design. Here, although the two courses belong to the same ecosystem, they become comparable according to the computational role of the instrument, i.e., either VPL or RTVE (Figure 8). It is important to underline that the results of Ecosystem II seem to have been negatively affected by low scores in Digital Heritage and Design. Despite the high scores of attractiveness, efficiency, dependability, stimulation, and novelty in Architectural Studio VI, the interaction with RTVE instruments, which is largely of Unity in this case, has an adverse impact on Ecosystem II.

### Contextuality

The results show that the digitalisation of design teaching requires student response and therefore contextual planning, no matter how advanced the tools and techniques are. Given that DDEs turn architectural studios into multidisciplinary environments. DDEs addressing what factors are efficient to design a novel andragogy landscape adds significance to studying teaching design in VR. Today, VR technologies may look very technical but it appears that design studios will adopt these techniques and accept them as built-in functions. Studying the formulation of an integrated VR infrastructure in architectural design education, this research indicates various advantages of virtual and digital design ecosystems over each other. But the contextuality aspect in an integrated VR infrastructure drives student-centered approaches for developing interesting and spreading the use of VR. The paper demonstrated how two ecosystems are alternative to each other while highlighting the role of the instructor that should be contextual to the computational aspects raised in every DDE. To understand the development of VR in design ecosystems, a combination of focus on both hardware and software solutions is required. Preventing the disintegration of VR from this hybrid view supports the engagement of design students with cutting-edge technologies.

## Conclusion

To design a new curriculum in undergraduate architecture education, it is important to take account of how well the studio environment and the student profile work together in symbiosis. Given that, the goal of this study is to conceive a novel andragogic landscape at Artuklu Architecture, in which the studio environment calibrates the student for being a self-directed learner. The breakout of the COVID-19 pandemic proved the importance of research on self-learning in higher education, particularly design-related courses once conducted face-to-face. Testing two alternative digital design ecosystems, the paper draws attention to online education with research results on teaching design via virtual environments. In addition to that researches on collaboration over the internet which is online education in design-related courses like Virtual Design Courses (VDSs) is the topic the CAD conferences as well as the results of this study addresses that.

This study offers insight into the current position of computational science in architecture education by examining how students interact with digital instruments in design studios. The motive behind the study lies in the growing role of VR in teaching architectural design to undergraduates. The paper first illustrates a brief development of the Virtual Reality (VR) concept within architectural design studios. Evolving from a variety of concepts of Virtual Design Studios (VDSs), Digital Design Ecosystems (DDEs) involve a combination of hardware and software instruments.

This paper illustrates the role of teaching architectural design in DDEs by demonstrating two settings, which are medium-oriented design processes (Ecosystem I) and content-oriented design processes (Ecosystem II). In Ecosystem I, students are exposed to a package of 3D tools for design, modeling, documentation, rendering, and animation, which are Blender, Rhinoceros, and Revit. In Ecosystem II, students engage with the computational and interaction aspects of Rhinoceros with Grasshopper and Unity. By that, studying Ecosystem II also gives insight on the use of visual programming languages (Grasshopper) and real-time virtual engines (Unity) in architectural education. The concentration on computational aspects greatly determines the shape of the design process and outcome in Ecosystem II as compared with which Ecosystem I is largely defined by the integration of 3D modeling techniques explicitly taught on specific tools. Both ecosystems are studied based on two undergraduate courses at the Department of Architecture, Mardin Artuklu University. Likewise, the intensity of interaction with VR tools in all of four courses varied in two incomparable, but connate, levels of qualities.

The implementation of a User Experience Questionnaire (UEQ) allowed what impacts specific DDEs can leave on different types of design interaction during design ideation and the creation of virtual environments. Student feedback from each course generated 4 different datasets. The use of the User Experience Questionnaire (UEQ) shows that students find DDEs “complicated” and “confusing” as well as stimulating and attractive. This study contributes to the robust planning of DDEs in design education. The findings suggest that the perspicuity aspects of student interaction in all DDEs bare the risk of being “complicated” and “confusing” software. The results demonstrate a conflict between task-related and non-task related qualities. DDEs are found attractive, stimulating, and original despite low scores about the pragmatic qualities of perspicuity, efficiency and dependability.

The research contributes to future work in the contextualization of the design teaching efforts using VR. The data obtained in this study will contribute to the robust planning of architectural design studios in architectural design education based on the use of VR, and other digital methods and technologies.

## Data Availability Statement

The datasets generated for this study are available on request to the corresponding author.

## Ethics Statement

Ethical review and approval was not required for the study on human participants in accordance with the local legislation and institutional requirements. The patients/participants provided their written informed consent to participate in this study.

## Author Contributions

The authors confirmed being the sole contributors of this work and approved it for publication.

## Conflict of Interest

The authors declare that the research was conducted in the absence of any commercial or financial relationships that could be construed as a potential conflict of interest. The handling editor declared a shared affiliation, though no other collaboration with one of the authors BA.

## Footnotes

^1^The Department of Architecture at the Mardin Artuklu University appears as Artuklu Architecture in the rest of the text. School website: http://www.artuklu.edu.tr/mimarlik-fakultesi. Also see the official social media accounts @artuklumimarlik on Instagram and @mimarlikartuklu on Twitter.
